# Inhibition activity of a traditional Chinese herbal formula Huang-Lian-Jie-Du-Tang and its major components found in its plasma profile on neuraminidase-1

**DOI:** 10.1038/s41598-017-15733-7

**Published:** 2017-11-14

**Authors:** Xuelin Zhou, Haotian Li, Zhilong Shi, Sijia Gao, Shizhang Wei, Kun Li, Jiabo Wang, Jianyu Li, Ruilin Wang, Man Gong, Yanling Zhao, Xiaohe Xiao

**Affiliations:** 1 Department of Pharmacy, 302 Military Hospital of China, Beijing, People’s Republic of China; 2 China Military Institute of Chinese Medicine, 302 Military Hospital of China, Beijing, People’s Republic of China; 3 Integrative Medical Center, 302 Military Hospital of China, Beijing, People’s Republic of China; 4 College of Pharmacy, Chengdu University of Traditional Chinese Medicine, Chengdu, People’s Republic of China

## Abstract

Huang-Lian-Jie-Du-Tang (HLJDT), a traditional formula with four TCM herbs, has been used for hundred years for different diseases. The current study aimed to assess the inhibitory activity of HLJDT against H1N1 neuraminidase (NA-1), and identify potent NA-1 inhibitors from its plasma profile. The *in vitro* NA-1 study has shown that the water extract of HLJDT potently inhibited NA-1 (IC_50_ = 112.6 μg/ml; Ki = 55.6 μg/ml) in a competitive mode. The IC_50_ values of the water extracts of its four herbs were as follows: Coptidis Rhizoma, 96.1 μg/ml; Phellodendri Chinensis Cortex, 108.6 μg/ml; Scutellariae Radix, 303.5 μg/ml; Gardeniae Fructus, 285.0 μg/ml. Thirteen compounds found in the plasma profile of HLJDT were also identified as potent NA-1 inhibitors, which included jatrorrhizine, palmatine, epiberberine, geniposide, oroxylin A, berberine, coptisine, baicalein, wogonoside, phellodendrine, wogonin, oroxylin A-7-O-glucuronide and baicalin (sorted in ascending order by their IC_50_ values). Their inhibitory activities were consistent with molecular docking analysis when considering crystallographic water molecules in the ligand-binding pocket of NA-1. Our current findings suggested that HLJDT can be used as a complementary medicine for H1N1 infection and its potent active compounds can be developed as NA-1 inhibitors.

## Introduction

Highly infectious influenza A virus is pandemics and recurrent annual epidemics, and causes severe respiratory illness and death, especially in the elderly, children, and weakness. Neuraminidase (NA), a surface glycoprotein antigen, is one of biomarkers for subtype classification of influenza A virus. NA facilitates the release of influenza A virus via hydrolyzing glycosidic linkages of terminal sialic acid residues, which is critical to infection progression in the host. Current treatment strategy for influenza virus infection is to inhibit NA function^1^. Several crystal structures of NA are obtained, and these structures facilitate structure-based drug discovery of NA inhibitors^1^. Two commercial drugs zanamivir (Relenza) and oseltamivir (Tamiflu), as derivatives of sialic acid, have been developed through this process. However, the supply of these drugs is limited. It is not possible to prescribe these drugs in the countryside of China when patients get influenza virus infection without serious symptoms. Therefore, it is necessary to discover new drug candidates for treating H1N1 infection. Currently, natural products (e.g. chlorogenic acid^2^, quercetin-7-O-glucoside^3^ and catechins^4^) are considerable resources for the discovery of NA inhibitors.

Huang-Lian-Jie-Du-Tang (HLJDT) is a traditional Chinese herbal formula used for hundred years, which consists of four herbs such as Coptidis Rhizoma (“Huang-Lian” in Chinese, HL), Scutellariae Radix (“Huang-Qin” in Chinese, HQ), Phellodendri Chinensis Cortex (“Huang-Bo” in Chinese, HB) and Gardeniae Fructus (“Zhi-Zi” in Chinese, ZZ) at the weight ratio of 3:2:2:3. It has been clinically used for treating sepsis^5^, inflammation^6^, cardiovascular diseases^7^, and Alzheimer’s disease^8^. After its oral administration, the major chemical components found in rat plasma have been identified, which include alkaloids (e.g. coptisine, berberine, and palmatine) and flavones (e.g. baicalein and wogonin)^9^.

**Figure 1:**
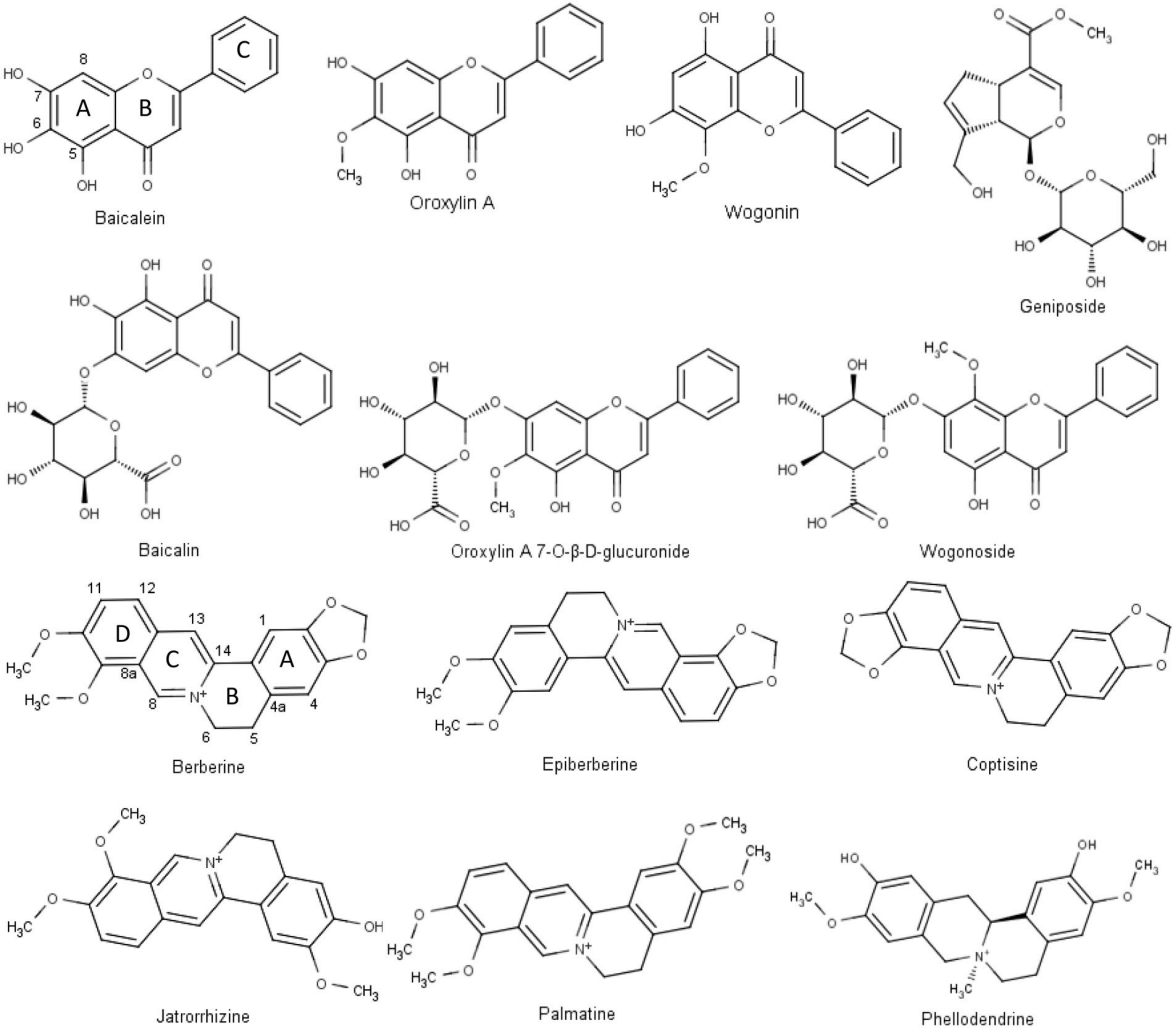
Structures of major chemical components found in the plasma profile of Huang-Lian-Jie-Du-Tang, as reported.

Although HLJDT is not traditionally used for the treatment of influenza A virus infection, some of its active components, such as baicalein^10^, berberine^11^ and coptisine^12^, have been identified as effective inhibitors of various NA subtypes. Other major components, detectable in the plasma profile of HLJDT, are supposed to be a potential resource for discovering NA inhibitors due to their similar structures. The aim of our current study was to evaluate the inhibitory activity of the water extracts of HLJDT and its four herbs on NA-1, and identify potent NA-1 inhibitors from its plasma profile (see chemical structures in Fig. 1) by *in vitro* inhibition study. Further, the inhibition of active compounds against NA-1 was also evaluated by *in silico* molecular simulation, which shows a better understanding for the binding mechanisms of the active compounds in ligand-binding pocket of NA-1. The results would provide information for further investigation on HLJDT as a complementary medicine in clinics for treating H1N1 infection, and its potent NA-1 inhibitors can also be a chemical resource for new drug development.

## Results and Discussion

### *In vitro* inhibition and enzymatic kinetic study of HLJDT

**Figure 2:**
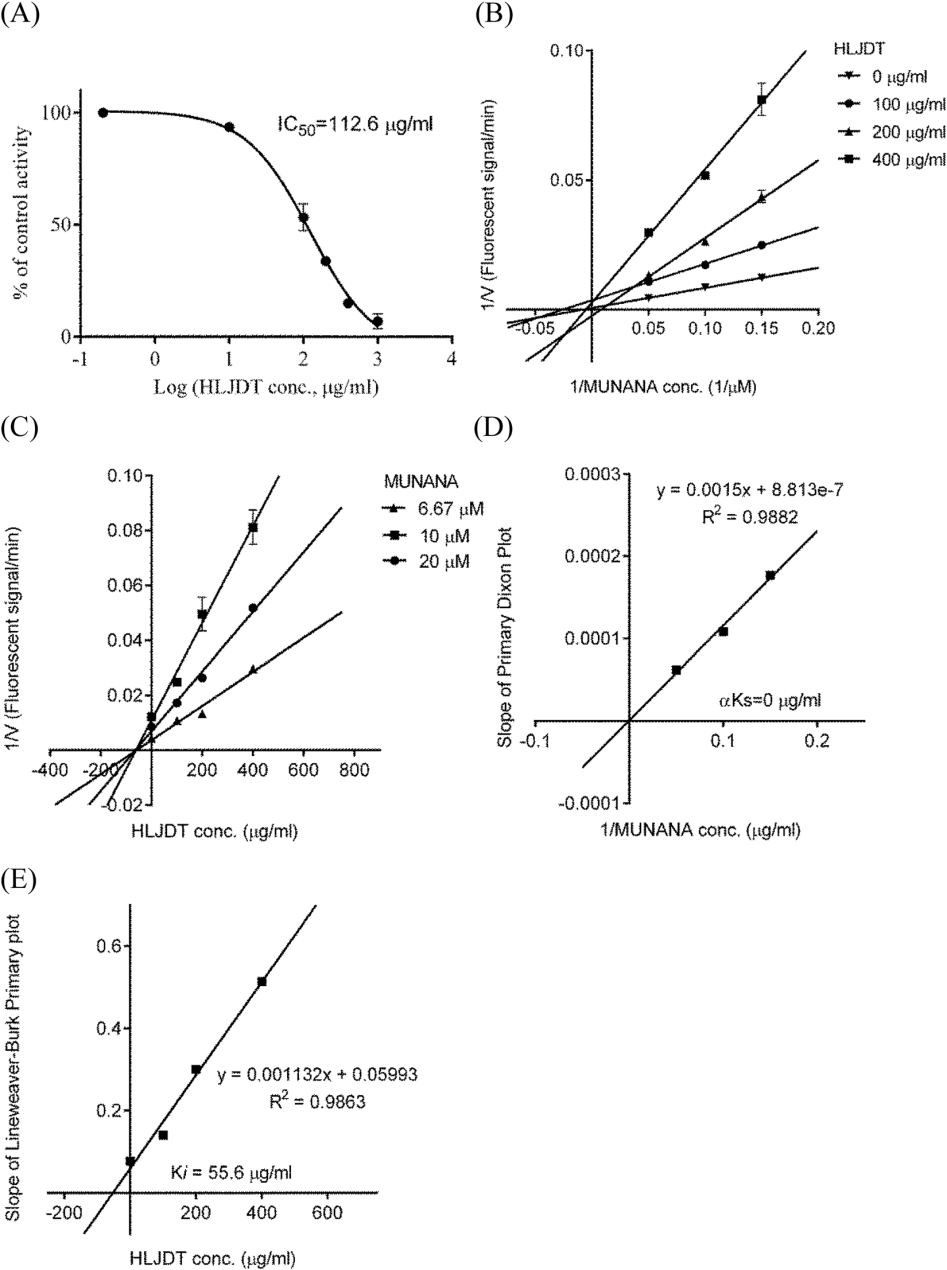
Inhibition value and mode of Huang-Lian-Jie-Du-Tang on neuraminidase-1 were obtained from (**A**) inhibition curve, (**B**) Primary Lineweaver–Burk plot, (**C**) Dixon plot, (**D**) Secondary Dixon plot and (**E**) Secondary Lineweaver-Burk plot for Ki (n = 3). HLJDT inhibited neuraminidase activity in competitive mode with IC_50_ value of 112.6 μg/ml and Ki value of 55.6 μg/ml.

When the substrate (MUNANA) concentration was 20 μM, the IC_50_ and IC_10_ values of HLJDT on NA-1 activity were about 112.6 ± 6.7 μg/ml and 19.3 ± 4.0 μg/ml, respectively (Fig. 2A). When compared to those of the positive NA inhibitor peramivir (IC_50_ = 478.8 ± 15.6 μg/ml; IC_10_ = 64.8 ± 8.4 μg/ml), HLJDT showed potent inhibition activity on NA-1.

For graphical inspection on the inhibition type of HLJDT, Primary Lineweaver-Burk plot (obtained by reciprocal of reaction velocities versus reciprocal of MUNANA’s concentrations) and Dixon plot (obtained by reciprocal of reaction velocities versus HLJDT’s concentrations) were firstly applied. As shown in Fig. 2B,C, the straight lines did not intersect on the x-axis or first quadrant in the Primary Lineweaver-Burk plot, but intersected on the x-axis in the Dixon plot. However, possibly due to experimental deviation, the inhibition type can not be confirmed by Primary Lineweaver-Burk plot and Dixon plot. For confirmation of the inhibition type, Secondary Dixon plot (obtained by the slopes of the regression lines in the Dixon plot versus reciprocal of MUNANA’s concentrations) was further drawn. As shown in Fig. 2D, the straight line goes through the origin, showing a competitive inhibition of HLJDT on NA activity^13^. Secondary Lineweaver-Burk plot for *K*_*i*_ (obtained by the slopes of the regression lines in the Primary Lineweaver-Burk plot versus HLJDT’s concentrations) in Fig. 2E showed that the *K*_*i*_ value of HLJDT on NA-1 activity was 55.6 μg/ml.

This is the first time to report NA-1 inhibition activity of HLJDT. Since HLJDT is a clinically used formula, it is very safe for TCM practitioners to prescribe it for patients with influenza-like symptom before prescribing western drugs like Tamiflu.

### *In vitro* inhibition study of each herb

**Figure 3:**
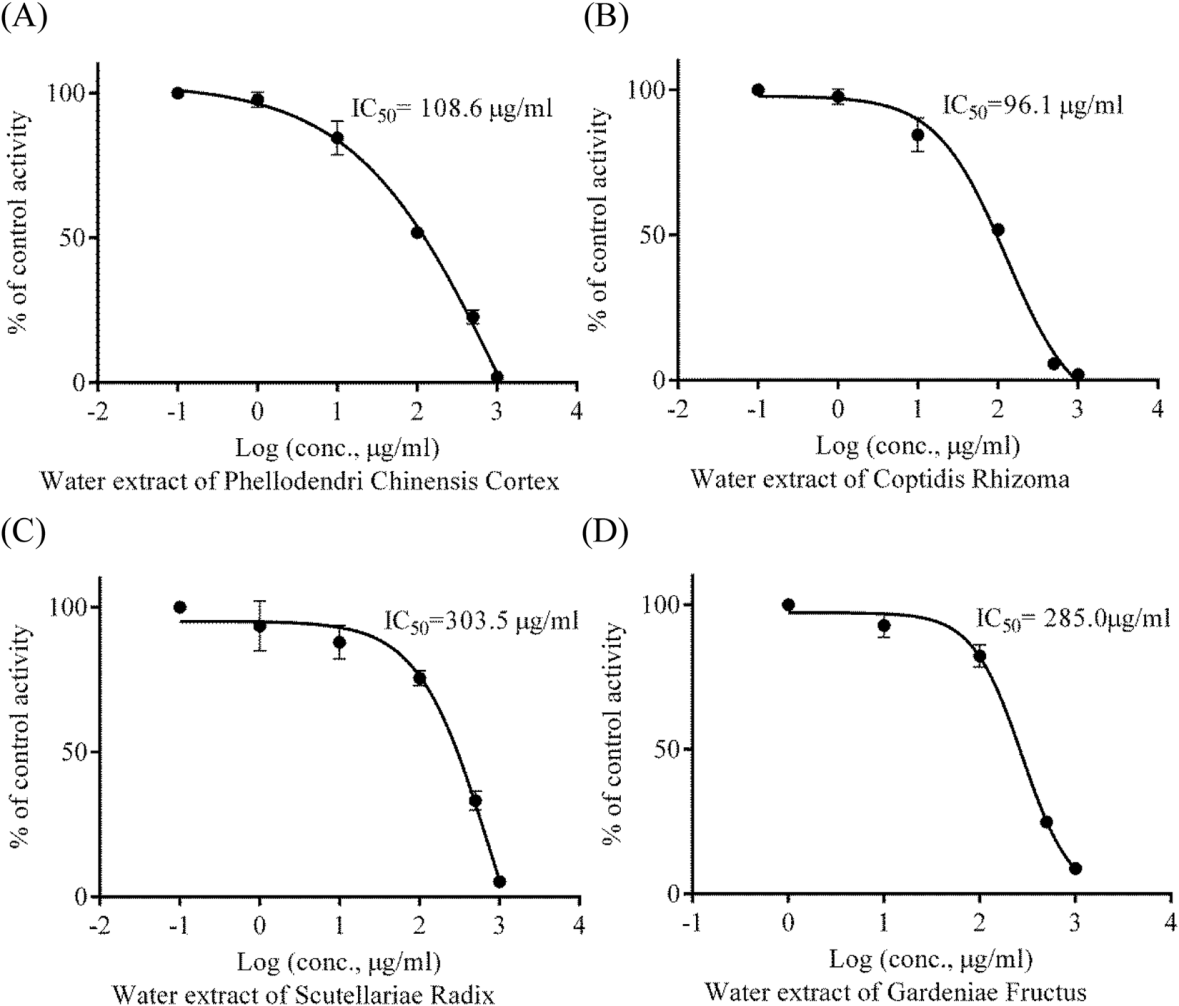
Inhibition curves of the water extracts of four herbs in Huang-Lian-Jie-Du-Tang, including (**A**) Phellodendri Chinensis Cortex, (**B**) Coptidis Rhizoma, (**C**) Scutellariae Radix and (**D**) Gardeniae Fructus, on neuraminidase-1 activity (n = 3).

In Fig. 3, when using MUNANA concentration at 20 μM, the IC_50_ and IC_10_ values of each herb on NA activity were as follows: Phellodendri Chinensis Cortex (HB), IC_50_ = 108.6 ± 8.6 μg/ml and IC_10_ = 8.4 ± 3.3 μg/ml; Coptidis Rhizoma (HL), IC_50_ = 96.1 ± 7.6 μg/ml and IC_10_ = 9.9 ± 1.1 μg/ml; Scutellariae Radix (HQ), IC_50_ = 303.5 ± 21.9 μg/ml and IC_10_ = 28.0 ± 8.7 μg/ml; Gardeniae Fructus (ZZ), IC_50_ = 285.0 ± 16.6 μg/ml and IC_10_ = 37.4 ± 2.7 μg/ml. According to One-way ANOVA with Tukey’s multiple comparisons post-test, there were no significant difference among the IC_50_ values of HB, HL and HLJDT (*P* > 0.05). Meanwhile, the IC_50_ value of HLJDT was significantly lower than the one of HQ and ZZ (*P* < 0.01). These suggested that HB and HL were the major ingredients of HLJDT responsible for NA-1 inhibition, and HQ and ZZ were also involved in NA-1 inhibition. Since the HLJDT formula was designed according to the TCM theory, its safety has been observed in its clinical application for hundreds of years. Its composition should not be changed in its application.

### *In vitro* inhibition study of active compounds in HLJDT

As shown in Table 1, all tested compounds showed inhibitory activities on NA-1. According to One-way ANOVA with Tukey’s multiple comparisons post-test, the tested compounds were sorted in ascending order by their IC_50_ values as follows: palmatine (17.8 ± 0.8 μg/ml) ~ jatrorrhizine (22.7 ± 0.2 μg/ml) > epiberberine (33.6 ± 0.3 μg/ml) ~ geniposide (34.0 ± 1.8 μg/ml) > oroxylin A (57.3 ± 6.3 μg/ml) > berberine (78.6 ± 5.5 μg/ml) > coptisine (104.6 ± 18.8 μg/ml) > baicalein (159.5 ± 13.4 μg/ml) > wogonoside (186.4 ± 5.6 μg/ml) > phellodendrine (205.1 ± 18.9 μg/ml) > wogonin (246.1 ± 10.3 μg/ml) > oroxylin A-7-O-glucuronide (286.1 ± 44.2 μg/ml) > peramivir (478.8 ± 15.6 μg/ml) > baicalin (735.2 ± 59.1 μg/ml). The IC_10_ values of alkaloids including epiberberine, jatrorrhizine, berberine, palmatine, coptisine, and geniposide were about 1.9~20 μg/ml (Table 1). In the pharmacokinetic study of HLJDT, the maximum plasma concentrations (Cmax) of these compounds were about 1~8 μg/ml^14,15^, indicating that HLJDT possibly has therapeutic effect on H1N1 infection according to Cmax values and NA-1 inhibition activities of these compounds.

**Table 1:** Inhibition values (Mean ± SEM) of neuraminidase-1 inhibitors from the plasma profile of Huang-Lian-Jie-Du-Tang (n = 3).

Compounds	IC_50_ (μg/ml)	IC_10_ (μg/ml)
Baicalein	159.5 ± 13.4	75.0 ± 29.1
Baicalin	735.2 ± 59.1	75.0 ± 29.1
Berberine	78.6 ± 5.5	3.1 ± 0.8
Coptisine	104.6 ± 18.8	14.0 ± 2.5
Epiberberine	33.6 ± 0.3	4.1 ± 0.2
Geniposide	34.0 ± 1.8	2.8 ± 0.2
Jatrorrhizine	22.7 ± 0.2	1.9 ± 0.1
Oroxylin A	57.3 ± 6.3	5.1 ± 1.4
Oroxylin A 7-O-β-D-glucuronide	286.1 ± 44.2	29.3 ± 7.8
Palmatine	17.8 ± 0.8	2.1 ± 0.1
Phellodendrine	205.1 ± 18.9	23.1 ± 2.7
Wogonin	246.1 ± 10.3	34.0 ± 5.1
Wogonoside	186.4 ± 5.6	19.6 ± 2.7
Peramivir^	478.8 ± 15.6	64.8 ± 8.4

^neuraminidase inhibitor, positive control.

The effects of baicalin,^16^ baicalein^10^ and berberine^11^ on H1N1 and NA-1 inhibition have been reported in the previous studies, which is consistent with the results found in our study. The therapeutic effect of geniposide on H1N1 virus has also been reported in H1N1-infected mice *in vivo* ^17^, and our study confirmed its NA inhibition activity. Palmatine, coptisine and jatrorrhizine inhibited the activity of bacterial NA^12^, so did wogonin and wogonoside on mouse liver sialidase^18^. However, it is still doubtful whether these five compounds can inhibit NA-1, since there are structural differences between NA-1 and these non-viral NA^19^. In our current study, the potent inhibition activities of geniposide, wogonin, wogonoside, palmatine, coptisine, and jatrorrhizine on NA-1 were confirmed. Moreover, it is the first time to report the inhibition effects of epiberberine, phellodendrine, oroxylin A, and oroxylin A 7-O-β-D-glucuronide on NA-1, which is possible due to their structural similarity to berberine or baicalein. Further, although their inhibition activities were not very high when they were at their respective maximum plasma concentrations, their accumulated inhibition effect should be considered to be responsible for the inhibition effect of HLJDT. As reported before^14^, the half-lives of the major compounds of HLJDT were about 240–480 min in the pharmacokinetic study, the administration frequency of HLJDT should be two or three times per day.

### Molecular docking analysis of potent NA-1 inhibitors

**Figure 4:**
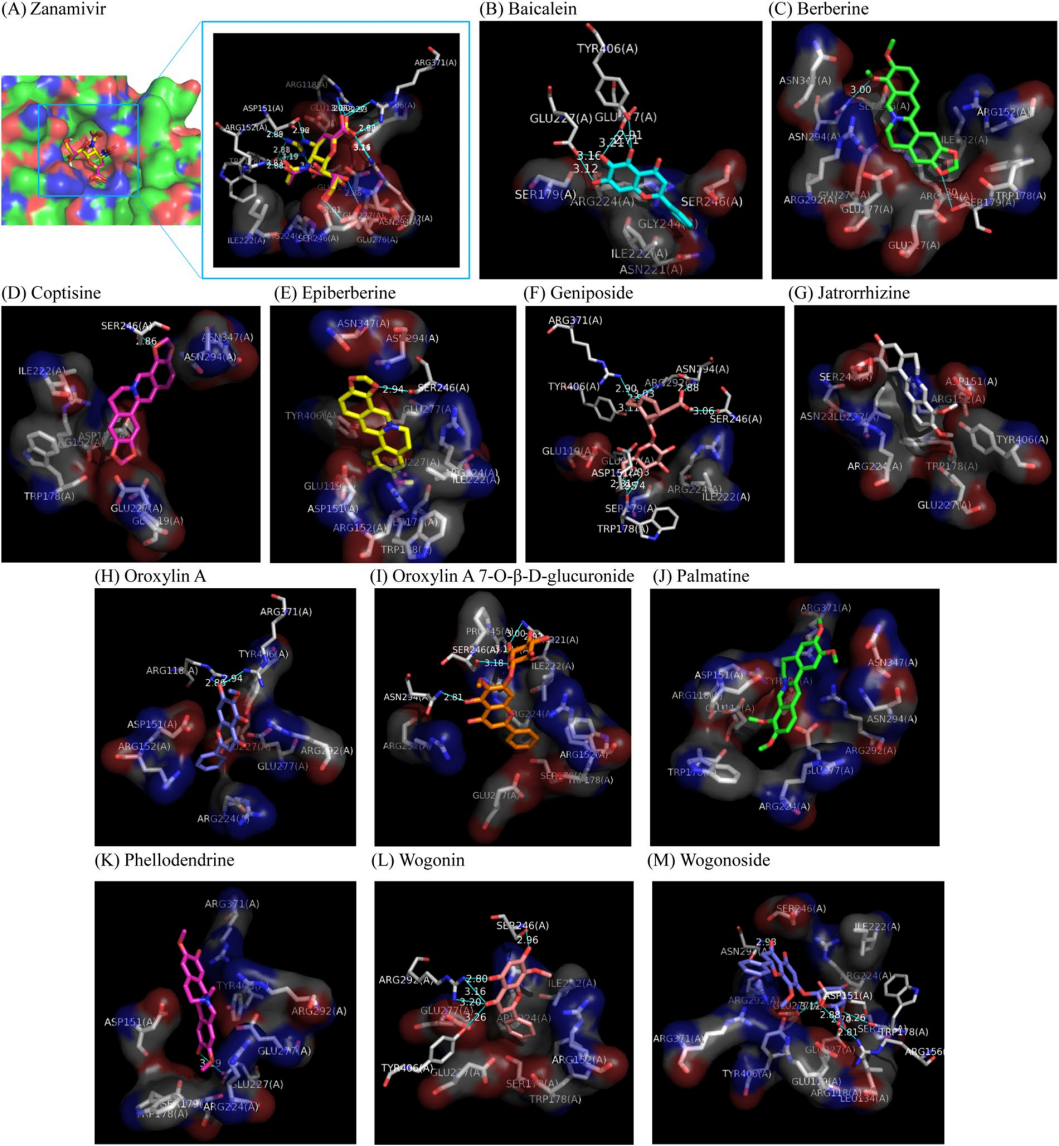
Molecular docking simulation shows the favorable binding positions of potent neuraminidase-1 inhibitors from the plasma profile of Huang-Lian-Jie-Du-Tang with lower binding free energy and proper distances (more than 1 Å) to water molecules in the ligand-binding cavity of H1N1 neuraminidase (PDB ID 3B7E). The 3D diagrams display the interactions of (**A**) zanamivir (re-docked pose, in purple sticks; crystallographic pose, in yellow sticks), (**B**) baicalein (in cyan sticks), (**C**) berberine (in green sticks), (**D**) Coptisine (in purple sticks), (**E**) Epiberberine (in yellow sticks), (**F**) Geniposide (in dull-red sticks), (**G**) Jatrorrhizine (in white sticks), (**H**) Oroxylin A (in blue sticks), (**I**) Oroxylin A 7-O-β-D-glucuronide (in ginger sticks), (**J**) Palmatine (in green sticks), (**K**) Phellodendrine (in purple sticks), (**L**) Wogonin (in dull-red sticks), and (**M**) Wogonoside (in blue sticks) to H1N1 neuraminidase with labeled amino residues responsible for generating binding free energy. Surfaces represent amino residues responsible for hydrophobic contacts with ligands. Blue lines with respective distances represent H-bonding between ligands and amino acid residues.

For the validation of docking procedure, zanamivir was firstly re-docked to its co-crystalized NA-1 structure (PDB ID 3B7E). As shown in the Fig. 4A, the pose of re-docked zanamivir (in purple sticks) was very close to the original crystallographic one (in yellow sticks) with a RMSD (Root-Mean-Square Deviation) of 0.285 Å which is much smaller than the cutoff of 2 Å. This indicated that the parameters used in the current procedures are accurate for molecular docking.

Current approach using Autodock Vina provided maximum 9 possible binding conformations of each compound in the ligand-binding pocket with different binding free energies, but this method ignored the existence of water molecules by removing water molecules at the beginning of docking procedures. The favorable conformations of zanamivir, jatrorrhizine and phellodendrine were observed with respective lowest binding free energies of −8.1, −6.8 and −7.4 kal/mol, and did not overlap the poses of crystallographic water molecules in the ligand-binding pocket of NA-1 (Table 2). However, the poses of other compounds with lowest binding free energies overlapped those of crystallographic water molecules in the ligand-binding pocket. Since the water molecule has a spherical volume with a radius of 1 Å, the binding conformations of each compound with lower binding free energies were re-selected with distance (more than 1 Å) to corresponding crystallographic waters^20^. As shown in Table 2, the binding free energy of the re-selected pose of sialic acid was −6.5 kal/mol.

**Table 2:** Logarithm of binding free energy (kal/mol) of active compounds from the plasma profile of Huang-Lian-Jie-Du-Tang in the ligand-binding pocket of H1N1 neuraminidase (PDB ID 3B7E) with or without water molecules.

Ligands	H1N1 without water molecules	H1N1 with water molecules
Baicalein	−8	−7.6
Baicalin	−8.8	—
Berberine	−7.4	−7.2
Coptisine	−8.5	−7.7
Epiberberine	−7.8	−6.9
Geniposide	−7.1	−7.1
Jatrorrhizine	−7.4	−7.4
Oroxylin A	−7.5	−7.4
Oroxylin A 7-O-β-D-glucuronide	−8.4	−7.7
Palmatine	−7.1	−6.8
Phellodendrine	−6.8	−6.8
Wogonin	−7.9	−7
Wogonoside	−8.5	−7.4
Sialic acid	−6.8	−6.5
MUNANA^#^	−7.1	—
Zanamivir*	−8.1	−8.1

^#^2-O-4-Methylumbelliferyl-4,7,8,9-tetra-O-acetyl-N-acetyl-alpha-D-neuraminic acid methyl ester, neuraminidase substrate; *neuraminidase inhibitor, positive control in the crystal structure; −: not observed.

Except baicalin and MUNANA, all other tested compounds bound to the ligand-binding pocket with lower binding free energies and appropriate distance to the corresponding crystallographic waters when compared to the one of sialic acid. As reported before, the 150 cavity, as part of the ligand-binding pocket, is closed when the inhibitor bound to the ligand-binding pocket of NA-1^1^. As such, the volume of NA-1 co-complexed with zanamivir (PDB ID 3B7E) used in the current study was smaller than the one of its native structure (PDB ID 3BEQ). This is may be the reason that there is no proper conformations of MUNANA and baicalin with bigger volumes in the ligand-binding pocket of NA-1 (PDB ID 3B7E).

### Molecular simulation of NA-1 inhibitors

As mentioned above, the binding conformations of active compounds with lower binding free energy and proper distances (more than 1 Å) to the water molecules in the ligand-binding cavities of NA-1 were selected, and simulated by both LigPlot + and PyMOL. Among these residues, eight charged and polar residues (e.g. Arg118, Asp151, Arg152, Arg224, Glu276, Arg292, Arg371 and Tyr406) directly interacted with the substrate in the ligand-binding cavities (the catalytic site) of NA-1^1^. As illustrated in Fig. 4A and summarized in Table 3, the binding conformation of zanamivir showed that as reported^1^, it directly interacted via Hydrogen-bonding with amino acid residues including Arg118, Asp151, Arg152, Trp178, Glu227, Glu276, Arg292, and Arg371, and through hydrophobic contacts with Glu119, Arg224, Ser246, Glu277, Asn294, and Tyr406, which contributed to the inhibition of zanamivir in the catalytic site. Similarly, inhibitors from the plasma profile of HLJDT bound to the catalytic site by interacting with these amino acid residues via H-bonding and/or hydrophobic contacts. These interactions took responsibility for the inhibition of these compounds on NA-1-mediated 4-MU formation.

**Table 3:** Binding conformation analysis of active compounds from the plasma profile of Huang-Lian-Jie-Du-Tang in the ligand-binding pocket of H1N1 neuraminidase (PDB ID 3B7E).

Ligands	H-bonding to amino acid residues (distance, Å)	Hydrophobic contacts to amino acid residues
Baicalein	Glu227 (3.1, 3.2 Å), Glu277 (2.7, 3.2 Å), Tyr406 (2.9 Å)	Ser179, Ile222, Asn221, Arg224, Gly244, Ser246
Baicalin	—	—
Berberine	Ser179 (3.3 Å), Asn294 (3 Å)	Arg152, Trp178, Ser179, Ile222, Arg224, Glu227, Glu276, Glu277, Ser246, Arg292, Asn294, Asn347
Coptisine	Ser246 (2.9 Å)	Glu119, Asp151, Arg152, Trp178, Ile222, Glu227, Asn294, Asn347
Epiberberine	Ser246 (2.9 Å)	Glu119, Asp151, Arg152, Ser179, Trp178, Ile222, Arg224, Glu227, Glu277, Asn294, Asn347, Tyr406
Geniposide	Asp151 (1.6 Å), Trp178 (1.9, 2.0 Å), Glu227 (2.0 Å), Ser246 (3.1 Å), Arg292 (1.9, 3.0 Å), Asn294 (1.7 Å), Arg371 (1.9 Å), Tyr406 (3.1 Å)	Glu119, Ser179, Ile222, Arg224
Jatrorrhizine	—	Asp151, Arg152, Trp178, Asn221, Ile222, Arg224, Glu227, Ser246, Tyr406,
Oroxylin A	Arg118 (3.9 Å), Arg371 (3.9 Å)	Arg152, Asp151, Arg224, Glu227, Glu277, Arg292, Tyr406
Oroxylin A 7-O-β-D-glucuronide	Asn221 (2.9, 3.0 Å), Gly244 (3.1 Å), Ser246 (3.2 Å), Asn294 (2.3 Å)	Arg152, Trp178, Ser179, Ile222, Arg224, Glu227, Pro245, Arg292
Palmatine	—	Arg118, Glu119, Asp151, Trp178, Arg224, Glu277, Arg292, Asn294, Asn347, Arg371, Tyr406
Phellodendrine	Glu227 (3.2 Å)	Asp151, Trp178, Ser179, Arg224, Glu277, Arg292, Arg371, Tyr406
Wogonin	Ser246 (4.0 Å), Arg292 (3.2, 3.2, 3.8 Å), Tyr406 (3.3 Å)	Arg152, Trp178, Ser179, Ile222, Arg224, Glu227, Glu277
Wogonoside	Asp151 (1.7 Å), Arg156 (1.6 Å), Trp178 (2.8, 3.2 Å), Glu227 (1.7 Å), Glu277 (3.1 Å), Asn294 (2.0 Å)	Arg118, Glu119, Leu134, Ser179, Ile222, Arg224, Ser246, Arg292, Arg371, Tyr406
Sialic acid	Arg118 (1.7 Å), Glu119 (1.7 Å), Asp151 (1.7, 2.7 Å), Trp178 (3.2 Å), Glu227 (1.9, 3.1 Å), Glu277 (1.9 Å), Arg292 (1.7, 3.2 Å), Arg371 (1.8, 3.1 Å), Tyr406 (1.8 Å)	Ile222, Arg224
Zanamivir*	Arg118 (2.0 Å), Asp151 (1.7, 2.0 Å), Arg152 (1.7 Å), Trp178 (1.5, 3.1 Å), Glu227 (3.1 Å), Glu276 (1.6, 1.6 Å), Arg292 (3.1, 3.2 Å), Arg371 (3.0, 3.1 Å)	Glu119, Arg224, Ser246, Glu277, Asn294, Tyr406

^*^neuraminidase inhibitor, positive control in the crystal structure; −: not observed.

Among these compounds, jatrorrhizine and palmatine interacted with amino acid residues in the catalytic site of NA-1 only through hydrophobic contacts. Besides hydrophobic contacts, other inhibitors tested in this study strongly interacted amino acid residues through hydrogen bonds. Hydroxyl groups at C-5, C-6 and C-7 of baicalein interacted with Glu227, Glu277 and Tyr406 via H-bonding. Hydroxyl group at C-7 of oroxylin A generated H-bonding with Arg118 and Arg371, while oroxylin A 7-O-β-D-glucuronide generated H-bondings via hydroxyl group at C-5 with Asn294, as well as its glucuronide with Asn221, Gly244, and Ser246. Wogonin formed H-bondings through ketone group at C-3 with Arg292 and Tyr406, hydroxyl group at C-5 with Arg292, and hydroxyl group at C-7 with Ser246. Likewise, wogonoside developed H-bondings through ketone group at C-3 with Asn294, and glucuronide with Asp151, Arg156, Trp178, Glu227, and Glu277. Methoxyl group at C-9 and 1,3-dioxolane at C-2, 3 of berberine interacted via H-bondings with Asn294 and Ser179, respectively, which were different from those of bacterial NA^12^. Ser246 formed H-bondings with 1,3-dioxolane at C-2, 3 of coptisine and epiberberine, hydroxyl group at C-2 of phellodendrine, respectively. Hydrogen bonds of geniposide were formed through hydroxymethyl group at C-7 with Arg292, Arg371 and Tyr406, 7-(hydroxymethyl)-methyl ester with Ser246, 4-carboxylic acid with Asn294, and glucosyl group with Glu227, Trp178 and Asp151.

### Structure-activity relationship

According to the inhibition activities (IC_50_ values) of tested compounds on NA-1, structure-activity relationships of flavones and isoquinoline alkaloids tested in the current study were explored and summarized as follows.

Among flavones, structures with a methoxyl group instead of hydroxyl group at C-6 could increase NA-1 inhibition. For instance, oroxylin A (IC_50_: 57.3 ± 6.3 μg/ml) and oroxylin A 7-O-β-D-glucuronide (IC_50_: 286.1 ± 44.2 μg/ml), containing a methoxyl group at C-6, showed stronger inhibitory activity than baicalein (IC_50_: 159.5 ± 13.4 μg/ml) and baicalin (IC_50_: 735.2 ± 59.1 μg/ml), respectively. The comparison of inhibitory effects on NA-1 between oroxylin A (IC_50_: 57.3 ± 6.3 μg/ml) and wogonin (IC_50_: 246.1 ± 10.3 μg/ml) indicated that compounds substituted with methoxyl group at C-6 instead of C-8 may increase NA-1 inhibition. Additionally, structures of these flavones with a glucuronic acid moiety at C-7 showed lower activity against NA-1. For example, NA-1 inhibition of baicalin (IC_50_: 735.2 ± 59.1 μg/ml) and oroxylin A 7-O-β-D-glucuronide (IC_50_: 286.1 ± 44.2 μg/ml) were lower than those of baicalein (IC_50_: 159.5 ± 13.4 μg/ml) and oroxylin A (IC_50_: 57.3 ± 6.3 μg/ml), respectively. However, wogonoside (IC_50_: 186.4 ± 5.6 μg/ml) with a substitution of glucuronic acid group at C-7 showed a better NA-1 inhibitory effect than wogonin (IC_50_: 246.1 ± 10.3 μg/ml), which remains to be further explored.

Among isoquinoline alkaloids, compounds substituted with free methoxyl group(s) instead of 1,3-dioxolane at C-2, 3 and C-9, 10 could increase NA-1 inhibition. For instance, NA-1 inhibition of berberine (IC_50_: 78.6 ± 5.5 μg/ml), epiberberine (IC_50_: 33.6 ± 0.3 μg/ml) and palmatine (IC_50_: 17.8 ± 0.8 μg/ml) were all better than that of coptisine (IC_50_: 104.6 ± 18.8 μg/ml). In addition, compound substituted with hydroxyl group at C-3 (jatrorrhizine; IC_50_: 22.7 ± 0.2 μg/ml) instead of methoxyl group at C-3 (palmatine; IC_50_: 17.8 ± 0.8 μg/ml) could not increase NA-1 inhibition. Finally, compounds with saturated aliphatic hydrocarbon groups at C-8 and C-13 and hydroxyl group at C-11 showed lower activity against NA-1. For example, phellodendrine had lower inhibitory effect than palmatine (IC_50_: 17.8 ± 0.8 μg/ml) and jatrorrhizine (IC_50_: 22.7 ± 0.2 μg/ml).

### *In silico* prediction for oral toxicity in rodents

The LD_50_ values for the compounds tested in this study are predicted by PROTOX and shown in Table 4. Jatrorrhizine and Palmatine showed more toxic with oral LD_50_ values of 410 mg/kg, and the oral LD50 values of other compounds were about 1000–5000 mg/kg with less toxicity. The prediction was with high accuracy (54–100%).

**Table 4:** *In silico* prediction for oral toxicity in rodents by PROTOX.

Compound	Predicted oral LD_50_ (mg/kg)	Prediction accuracy
Baicalein	3919	71%
Baicalin	5000	69%
Berberine	1000	100%
Coptisine	1000	73%
Epiberberine	1000	73%
Geniposide	2000	71%
Jatrorrhizine	410	69%
Oroxylin A	4000	71%
Oroxylin A 7-O-β-D-glucuronide	5000	69%
Palmatine	410	69%
Phellodendrine	2803	73%
Wogonin	3919	100%
Wogonoside	5000	69%
Zanamivir	5000	54%

## Conclusion

This is the first time to report the NA-1 inhibition activity of HLJDT as a ready-to-use potent agent for anti-H1N1 infection, and it is suggested that it is valuable for TCM practitioners to use HLJDT and evaluate its efficacy in patients with influenza-like symptoms when they can not be diagnosed at the beginning. The *in vitro* inhibition activities of 13 compounds found in the plasma profile of HLJDT should be responsible for the possible therapeutic effect of HLJDT due to the correlation of their Cmax and NA-1 inhibition activity. Besides, it is the first time to report the inhibition effects of epiberberine, oroxylin A, oroxylin A 7-O-β-D-glucuronide, and phellodendrine on NA-1 *in vitro* and *in silico*.

## Materials and Methods

### Materials

All authentic standards (purity ≥ 98%) were purchased from ChromaBio Biotechnology (Chengdu, China). The screening kit for H1N1 neuraminidase inhibitors was provided by Beyotime Institute of Biotechnology (Shanghai, China). All other unspecified chemicals and reagents were supplied by Sigma (St. Louis, MO, USA).

### Herbal extraction

Scutellariae Radix (Lot No. 160562), Coptidis Rhizoma (Lot No. 160374), Phellodendri Chinensis Cortex (Lot No. 160528) and Gardeniae Fructus (Lot No. 160605) were purchased from Yan-He-Ling Pharmaceutical Company (Bejing, China). Raw herbs were authenticated by the supplier through thin layer chromatography and HPLC with authentic compounds according to Chinese Pharmacopoeia version 2015. Their voucher specimens were deposited at the Military Institute of Chinese Medicine, 302 Military Hospital, Beijing, China.

Raw herbs for the formula (containing Coptidis Rhizoma, 9 g; Phellodendri Chinensis Cortex, 9 g; Scutellariae Radix, 6 g; and Gardeniae Fructus, 6 g) were extracted by boiling with water (300 mL) for 60 min. After filtered, the residue was extracted again in the same way for another hour. The supernatant was mixed and subjected to freeze-dry. The dried extract was kept in the desiccator before use. The dried yield of HLJDT extract was ~31%. Each herb with respective amount in the formula was individually prepared by the same extraction method with 300 mL of water, and their extraction yields were as follows: Scutellariae Radix, ~55%; Coptidis Rhizoma, ~22.6%; Phellodendri Chinensis Cortex, ~24.6%; Gardeniae Fructus, ~35.4%.

### *In vitro* inhibition study

The inhibition study was performed with modification according to the manufacture’s instructions. Briefly, the inhibitor or extracts/compounds tested was mixed with recombinant NA-1, and then the substrate 2-O-4-Methylumbelliferyl-4,7,8,9-tetra-O-acetyl-N-acetyl-alpha-D-neuraminic acid methyl ester (MUNANA, 20 μM) was added for 1-hour enzymatic reaction. After that, 200 μL of stopping solution was added for the termination of reaction. The formed metabolite 4-methylumbelliferone (4-MU) was measured by BioTek Synergy H1 florescent plate reader (Winooski, VT, USA) with excitation wavelength at 355 nm and emission wavelength at 460 nm. DMSO (0.1%) served as vehicle control. The inhibition rate was calculated as follows.

$${\rm{Inhibition}}\,\mathrm{rate}( \% )=({{\rm{A}}}_{{\rm{vehicle}}{\rm{control}}}-{{\rm{A}}}_{{\rm{compound}}})/{{\rm{A}}}_{{\rm{vehicle}}{\rm{control}}}\times 100 \% .$$

### Enzymatic kinetic study

For enzymatic reaction, the inhibition constant (*K*_*i*_) values and inhibition types were measured by using three concentrations (6.67, 10 and 20 μM) of substrates and four concentrations of HLJDT (0, 100, 200, and 400 μg/ml). Other procedures were performed as mentioned above.

### Molecular docking study for NA inhibitors

Molecular docking analysis was preliminarily used to screen compounds from the plasma profile of HLJDT by using the software AutoDock Vina v.1.0.2^21^. The crystal structures of A/Brevig Mission/1/1918 H1N1 NA complexed with zanamivir (PBD ID 3B7E) were obtained from the Protein Data Bank^1^. The docking parameters were set with default values. The sizes of grid boxes were set as 20 Å × 20 Å × 20 Å for encompassing the ligand-binding pocket. Zanamivir was used as positive control.

The binding modes of active compounds with lower binding free energy and proper distances (more than 1 Å) to water molecules in the ligand-binding cavity of NA were chosen for further analysis of docking conformation. The 2D and 3D simulation results were illustrated by LigPlot + v.1.4.5 (http://www.ebi.ac.uk/thornton-srv/software/LIGPLOT/)^22^ and PyMOL Molecular Graphics System v.1.3 (Schrödinger, LLC, New York City, USA), respectively.

### *In silico* prediction for oral toxicity in rodents

PROTOX, a webserver for predicting oral toxicities of small molecules in rodents, was used for evaluating the oral toxicity of the compounds tested in this study^23^. The structures of the tested compounds were uploaded in the website, and results were generated by the protein–ligand-based pharmacophore models with the in-house toxicity database.

### Data analysis

Data were expressed as mean ± standard error of Mean (SEM). IC_50_ value was calculated by non-linear regression analysis with Prism version 5.0 (GraphPad Software, CA, USA). The inhibition constants (*K*_*i*_) and modes of different inhibitors to neuraminidase-1 were measured by graphical inspection from different plots (e.g. Primary Lineweaver-Burk Plot, Dixon plot, and Secondary Lineweaver-Burk plot for *Ki*) according to the previous report^13,24^. Data were analyzed by One-way ANOVA with Tukey’s multiple comparisons post-test. A *p* value less than 0.05 was considered statistically significant.
